# An active metasurface for field-localizing wireless power transfer using dynamically reconfigurable cavities

**DOI:** 10.1038/s41598-019-48253-7

**Published:** 2019-08-13

**Authors:** A. L. A. K. Ranaweera, Thanh Son Pham, Huu Nguyen Bui, Viet Ngo, Jong-Wook Lee

**Affiliations:** Department of Electronics Engineering, Information and Communication System-on-chip (SoC) Research Center, Kyung Hee University, Yongin, 17104 Republic of Korea

**Keywords:** Power distribution, Electronic and spintronic devices

## Abstract

Wireless power transfer (WPT) provides a convenient method of delivering energy to multiple devices. With the increasing use of WPT, safety concerns inevitably create the need for a reliable control mechanism. Previous approaches in advanced WPT or metamaterial-enhanced WPT, however, have the limitation that neither the intensity nor the shape of the field-localizing area can be dynamically controlled. To address this limitation, we introduce the novel concept of a hotspot or power-focused region using field-localizing WPT. Using the proposed method, we provide experimental evidence demonstrating that the location, shape, and intensity of the hotspot can be manipulated as desired. The hotspot effectively enhances power delivery to the intended device while reducing leakage to unwanted areas. To dynamically reconfigure the hotspots, we propose an active metasurface with multi-functionality due to its frequency switching and tuning capability. The dynamic reconfiguring capability provides a wide range of versatile practical applications, overcoming the limitations associated with passive metamaterials. Because the location, shape, and intensity of hotspots can readily be controlled, the proposed method is not limited to WPT applications. It can also be used for a broad range of applications that require precise control of power delivery.

## Introduction

Wave propagation has played a vital role in transferring and using energy throughout the evolution of human civilization. The scientific study of wave propagation control originated with attempts to control light. By successfully controlling light propagation at unwanted frequencies, photonic crystals have enabled remarkable advances in photonics^1–3^.

Another breakthrough in wave propagation control appeared with the realization of artificial materials called *metamaterials*^4^. The experimental studies^5–7^ of Pendry and Smith demonstrated wave propagation control through various applications of metamaterials^8^. Imaging immediately found innovative applications for metamaterials: overcoming the resolution limit beyond classical optics to search for a perfect lens^9^. In addition to imaging, metamaterials have shown great potential in applications such as beam shaping, steering, cloaking, and focusing. Using metamaterials, wave propagation control that can beat the diffraction limit and be focused into subwavelengths has been demonstrated with excellent results^10–13^.

Recently, applications of metamaterials have been extended to wireless power transfer (WPT). After Tesla’s pioneering work, research on WPT began to emerge from a group of researchers at MIT^14^. After a decade of experimentation, WPT has been finding many applications ranging from implantable medical devices^15,16^ to electric vehicles, with power ranging from microwatts to hundreds of watts. Furthermore, researchers have applied metamaterials to evanescent wave amplification; this approach successfully demonstrated enhanced WPT efficiency at extended distances^17–20^. Recently, a metasurface-based smart table using spatially localized surface wave was proposed for WPT^21^.

WPT is a convenient way to provide energy to multiple devices simultaneously^22^. Safely reaping the benefits of WPT in practical applications requires that unnecessary power leakage is kept below a certain level. Controlling the power leakage to unwanted areas, therefore, is as important as achieving high efficiency. Previously, leakage control has been achieved using non-periodic arrays by tailoring the electromagnetic near field^23^. Magnetic field shielding is also investigated using an anisotropic metamaterial slab with near-zero permeability^24^. Improving both the safety and efficiency of WPT can be effectively realized using selective field localization, which enables the provision of power to only an intended zone. Therefore, there is a great demand for selective and controlled power focusing on only an intended zone.

Various methods for localizing electromagnetic (EM) fields into the subwavelength region have been investigated. Previous approaches have used positional disorder at the structural level^25^, phase tuning of the incident beam^26^, and cavity mode resonance^27–30^. These previous studies, however, used passive metamaterials that do not allow tuning of their characteristics. Therefore, neither the intensity nor the shape of the field localization area can be controlled. Active metamaterials have been investigated to extend their functionality, but those efforts have focused mainly on optics and wireless communication^31–33^. Incorporating active metamaterials for WPT paves the way for applications that require dynamic reconfiguration, as does wave propagation control for field localization.

Motivated by these observations, we propose an active metasurface suitable for dynamically localizing fields into the subwavelength region. By focusing on the low-loss realization of a metamaterial, we present a field-focusing WPT system that provides controlled and selective power transmission into the intended zone. The power focusing region, which we call a *hotspot*, is realized using defect cavities created on the metasurface. The location, shape, and intensity of the hotspots are precisely and dynamically controlled. By focusing the fields on only the region of interest, both safety and efficiency are enhanced. Furthermore, we create several types of wireless power routing paths by reconfiguring a line of hotspots. Using the proposed method, we successfully transpose the use of the active metasurface into the WPT domain. Detailed explanations of each demonstration are given in the Results section.

## Results

### Active metasurface for wireless power transfer

**Figure 1:**
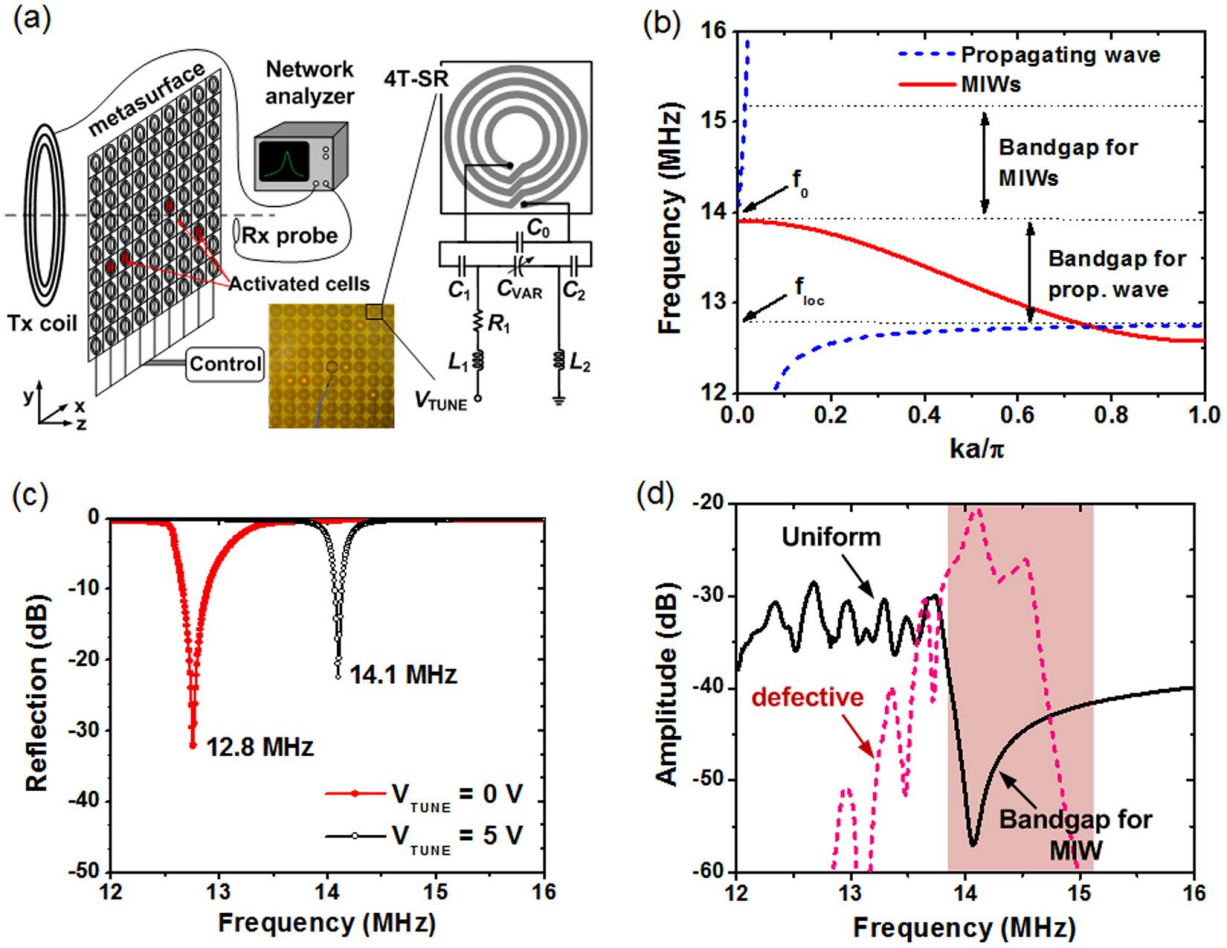
(**a**) A schematic and photo of the active metasurface with the unit cell, which has a tuning function. The distance between the Tx coil (Rx probe) and metasurface is 20 cm (5 cm). (**b**) Dispersion characteristics of MIWs and propagating waves. (**c**) Measured reflection of a single unit cell. (**d**) Transmission for the two cases of uniform and defective metasurface measured using a Tx coil and an Rx probe.

Figure 1 shows the proposed active metasurface used to achieve field-focusing WPT. The metasurface consists of 9 × 9 arrays of unit cells that are controlled individually. We shrink the bulky three-dimensional (3D) structure used in a previous study^34^ into a planar two-dimensional (2D) structure, which significantly reduces the loss associated with metallic inclusions in the unit cells. Each unit cell consists of a four-turn spiral resonator (4T-SR) and tuning circuitry. A detailed explanation of the metamaterial design and fabrication is provided in the Methods section.

The unit cell should achieve low MHz operation in a compact area. To embed the active function in the metasurface, we tested several unit cells with tuning functions^35^. Some unit cells consumed relatively high power. Some tested designs showed multiple resonances and exhibited insufficient resonance frequency splitting. Other designs lacked the ability to completely deactivate the resonance at the desired frequency. Figure 1(a) shows the proposed tunable unit cell. This structure has several advantages: simple fabrication, a relatively high *Q*-factor (~150 at 14.1 MHz), negligible power dissipation, a relatively wide tuning range, non-bianisotropic property, and good isolation (5.1 MΩ at DC) between adjacent unit cells. In addition, this unit cell exhibits distinct resonance switching between two widely separated frequencies.

Figure 1(b) shows the dispersion characteristics investigated in this work. When propagating waves interact with the metasurface, a surface wave (a guided mode) is formed below a local resonance frequency *f*_loc_, and its dispersion is shown as a dashed blue line^36^. Propagation is allowed in two regions: from zero to the lower band edge (*f*  < *f*_loc_) and the passband (*f* > *f*_0_) located above the bandgap. A bandgap exists between *f*_loc_ = 12.8 MHz and *f*_0_ = 14.1 MHz.

The situation is changed in the near-field regime, where a Tx coil is coupled to the metasurface: interestingly, the bandgap for the propagating waves allows magneto-inductive waves (MIWs) to propagate. The MIW is a kind of slow wave (its velocity is less than the speed of light) created by inter-element coupling between periodically arranged unit cells^37^. MIWs travelling along the array elements support backward propagation in the resonant frequency band. The dispersion of the MIWs is shown as a solid red line in Fig. 1(b). The MIWs have a passband below *f*_0_, which is a bandgap for the propagating waves. When the loss increases toward the band edges, the MIWs attenuate quickly at frequencies above *f*_0_^28^. In the upper region of the MIW passband, there is a deep hybridization bandgap whose origin is related to Fano interference. For more details, see the Discussion section.

Using the proposed active metasurface, transmission control is realized by simply switching the resonance frequency of the unit cell. Figure 1(c) shows the measured reflection coefficient |*S*_11_| of a single unit cell, which exhibits two distinct resonant frequencies, *f*_loc_ and *f*_0_. When the unit cell is turned off (*V*_TUNE_ = 0 V), there is a passband at around *f*_loc_, and a stopband is created at around *f*_0_. The locations of the two bands are interchanged when the cell is turned on (*V*_TUNE_ = 5 V) and resonates at *f*_0_. These results demonstrate the complete activation of resonance at two distinct frequencies, providing excellent switching capability realized by the active metasurface, which uses these two bands to control the transmission.

The physical mechanism for field-focusing is based on the unique combination of Fano interference and MIW propagation. We characterize the bands for propagation control using the transmission as a function of frequency for the case when a Tx coil and an Rx probe are used. Figure 1(d) shows the measured transmission for two cases. For Case 1, we characterize a uniform metasurface. All the array elements on the metasurface are turned off and resonate at *f*_loc_. This frequency, which is close to *f*_0_, is chosen because it allows strong field localization by inhibiting MIW propagation^38^. Then, the transmission shows the asymmetric shape of the hybridization bandgap^29^, which indicates the presence of Fano-type interference (the interference between discrete resonance and continuous fields). The hybridization bandgap is caused by zero transmission, which is attributed to complete destructive Fano interference. For Case 2, we characterize a metasurface with a defect cavity or a *defective metasurface*. All unit cells resonate at *f*_loc_ except one unit cell in the center, which is turned on via resonating at *f*_0_. The result shows a transmission peak at around *f*_0_. At the location of the surrounding unit cells (other than the defect cavity), surface wave propagation is prevented by the bandgap. This results in field confinement to the region of the cavity. The cavity mode (or trapped mode) is a special form of MIW that is confined to the cavity region. Using metallic wires as a resonating element, similar results were previously reported^29^. The result shows that the metamaterial can be engineered to create field localization in a cavity by blue-shifting the resonant frequency.

### Selective hotspot creation

**Figure 2:**
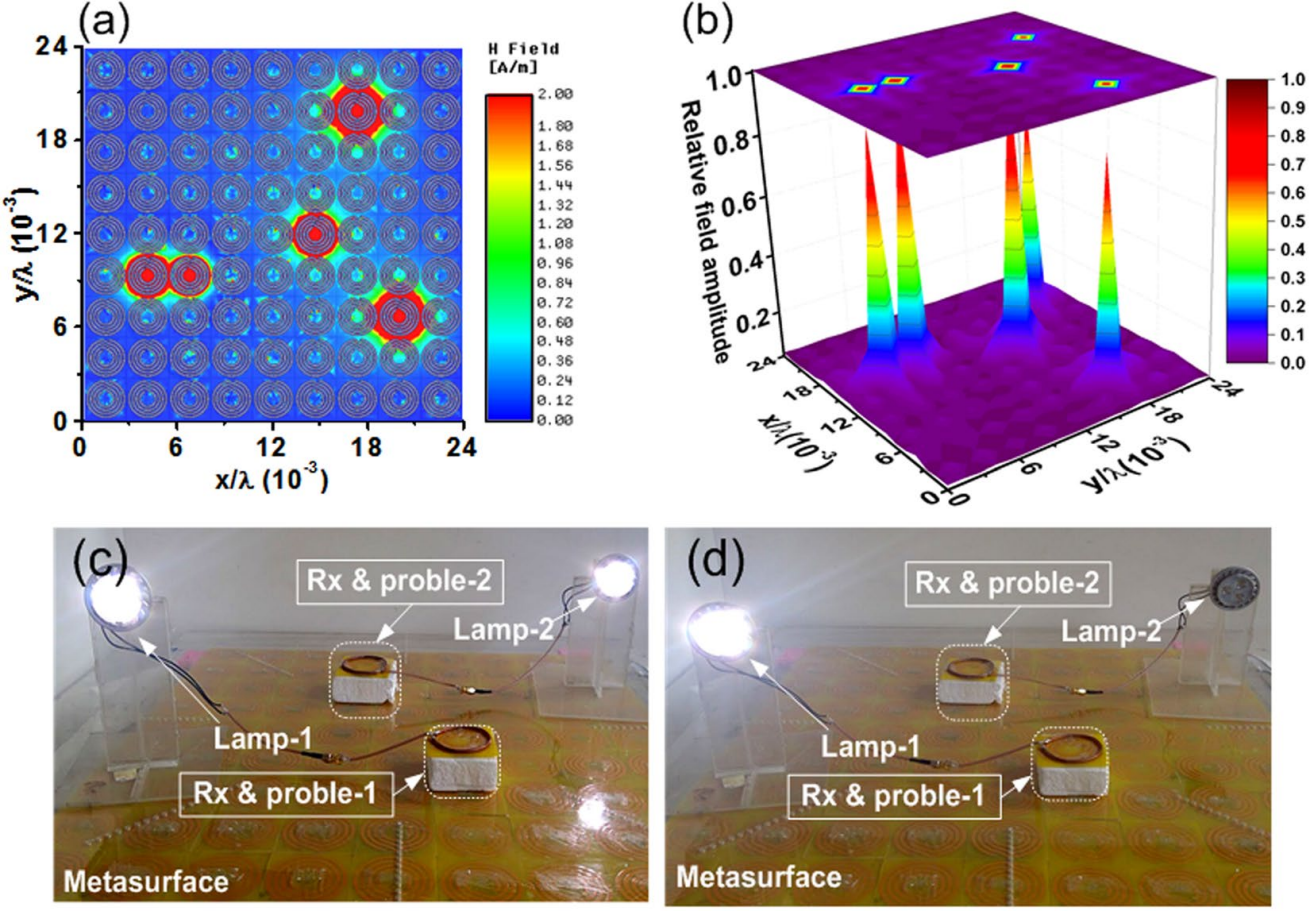
(**a**) Simulated field intensity distribution on metasurface, (**b**) measured field amplitude on a relative scale. Experimental demonstration of WPT to (**c**) two receivers, (**d**) single selected receiver. Each LED lamp (JKLcom MR16) consumes 4 W at 12 V.

Cavities on the metasurface form a defective metasurface. To investigate a defective metasurface, we use an EM simulator. Figure 2(a) shows the field intensity distribution on a metasurface (dimension normalized to wavelength λ). The region of the cavities shows strong field localization (a hotspot), and the field is relatively low and uniform outside the cavities. The result shows that the defective metasurface localizes the field on the subwavelength scale with a high contrast^38^. Using a small probe to scan the metasurface, we measure the field distribution. Field plots are obtained using the procedure described in the Methods section. Figure 2(b) shows the normalized field amplitude on the metasurface with its surface projection on top. The result clearly identifies the hotspots as sharp elevations from the base region. The Purcell factor can be used to quantify energy coupling to a cavity mode^39^ and thereby the field localization capability. We obtain the Purcell factor as 6.58 × 10^7^, which indicates that the defective metasurface can create a cavity with strong field localization. For more details, see the Discussion section. Using a selective hotspot, we demonstrate WPT to multiple receivers. Figure 2(c,d) show the experimental results using the active metasurface and light-emitting diode (LED) lamps. To allow efficient operation, we use a resonator coupling in which a set of Tx and Rx resonators are added close to the Tx coil and Rx probe, respectively. In the next section, we characterize the power transfer efficiency of the experiment.

The field localization enhances power transfer efficiency for a small receiver and reduces leakage power to unintended zones. A small receiver is used in various WPT applications. For example, a tiny receiver is required for implantable devices to facilitate a minimally invasive operation. In our experimental setup shown in Fig. 2, the size ratio between the Tx resonator and Rx probe is 16:1. In conventional WPT, the relatively large size ratio between Tx and Rx typically results in low efficiency. Using field localization, we demonstrate significant efficiency improvement.

**Figure 3:**
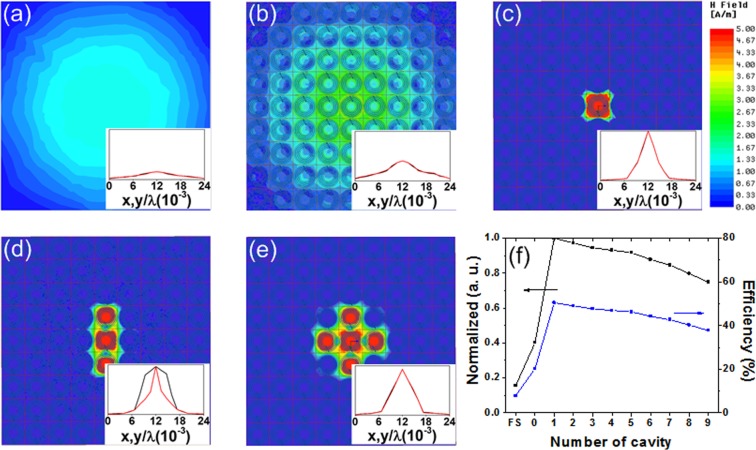
Comparison of field distribution for the case of (**a**) free space, (**b**) uniform metasurface without a cavity, and defective metasurface with (**c**) one cavity, (**d**) three cavities, (**e**) five cavities. The inset of each figure shows the relative transmissions along the *x-* and *y*-axes. (**f**) Normalized power transmission and efficiency as a function of the number of cavities.

Figure 3(a,b) show the field intensity for free space and a uniform metasurface, respectively. In the case of free space, the intensity is higher in the center than at the edge due to the incident field from the Tx resonator. In the center region, the uniform metasurface shows a relatively high intensity compared to the free space. In Fig. 3(c–e), unit cells are selectively activated in the center of the metasurface, thus creating various defect cavities. Figure 3(c) shows the field intensity where a single cavity is created. The result shows remarkable field localization at the center region. When a small probe is coupled to that cavity location, this strong localization allows highly efficient collection of power from the metasurface. Furthermore, the region of field localization can be shaped as desired, as shown in Fig. 3(c–e). The shape control allows selective WPT where power is focused only on an intended zone.

The insets in Fig. 3(a–e) show the relative power transmission ratio ($|{S}_{21}{|}_{x,y}^{2}/|{S}_{21}{|}_{max}^{2}$) along the *x*- and *y*-axes. The maximum power transmission ($|{S}_{21}{|}_{max}^{2}$) is achieved at the center of a metasurface with one cavity. Compared with the free space, the transmission is increased when a uniform metasurface is used. When a defective metasurface is used, the transmission is further increased at the center and reduced at the edge. This result indicates that the enhanced field localization achieved by the defective metasurface reduces the power leakage to the unintended region. The result is further confirmed using the power transmission, which is normalized using the highest power transfer ratio achievable by using one cavity. On the left axis of Fig. 3(f), we show the normalized transmission as a function of the number of cavities. These values are 0.16 and 0.4 for free space (FS) and uniform metasurface (zero cavities), respectively. The results show that the defective metasurface significantly increases the power transmission compared with both free space and the uniform metasurface. The ratio gradually decreases as the number of cavities increases, indicating the spread of power into a larger area. On the right axis of Fig. 3(f), we show the measured efficiency. The efficiency is evaluated using the transmission coefficient *S*_21_ similar to those reported in previous work^18^. The results show increasing efficiency up to one cavity and a gradual decrease as the number of cavities increases. Using the size ratio of 16:1 between a Tx resonator and Rx probe, a peak efficiency of 50.4% is achieved when one cavity is used. That result is significantly higher than the efficiency of free space (7.8%) or a uniform metasurface (20.3%). This corresponds to efficiency enhancements of 6.4× and 2.5×, respectively. These results show that efficiency is significantly increased using a defective metasurface.

**Figure 4:**
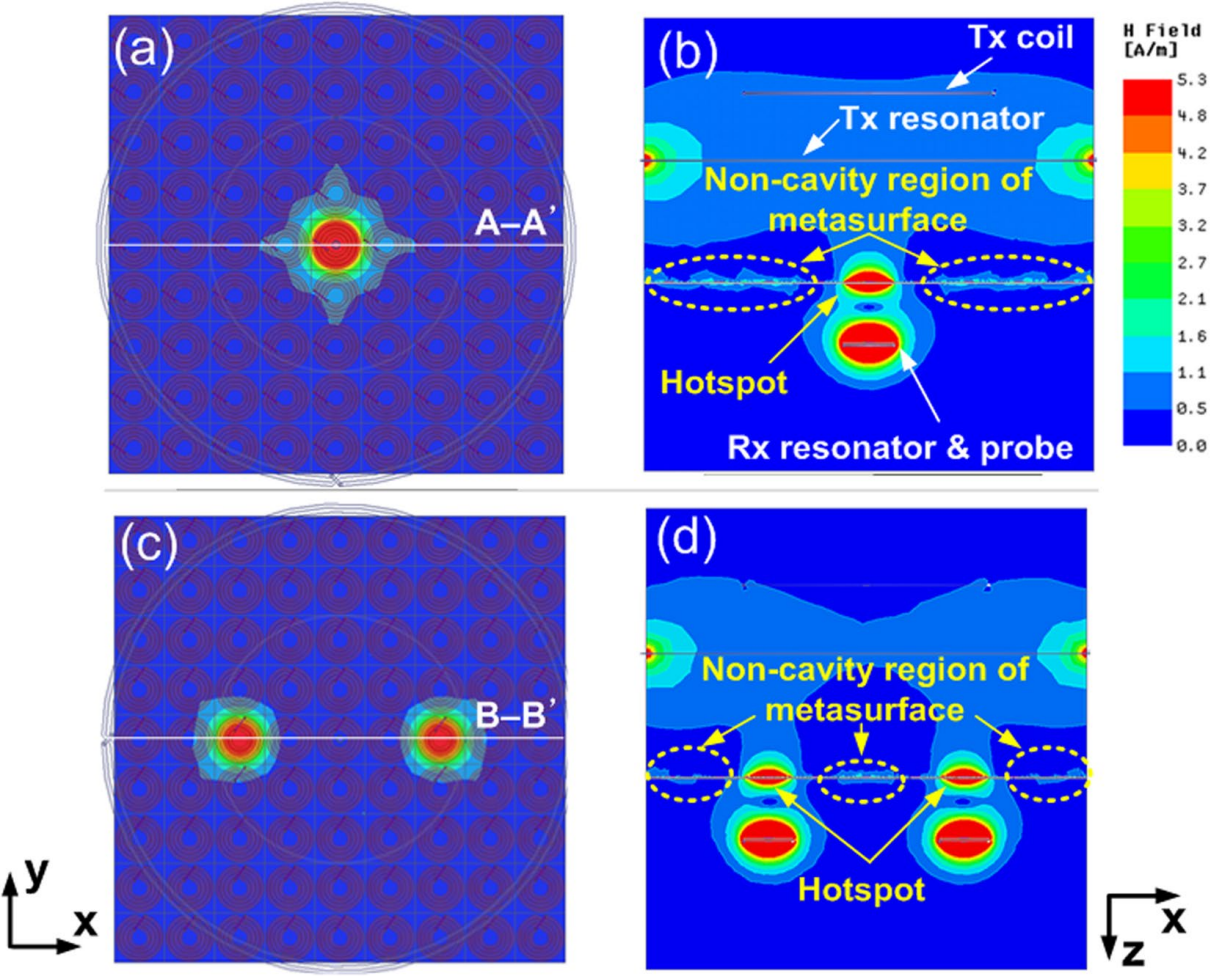
Field intensity distribution for the case of the defective metasurface with. (**a**) one cavity, (**b**) field intensity along A-A’, (**c**) two cavities, (**d**) field intensity along B-B’.

In addition to efficiency, one important consideration is the leakage power reflected by the metasurface creating a hotspot, which raises health concerns. Even though the transmitter is well matched (|*S*_11_|  < −10 dB), this does not mean that there is no power reflected by the metasurface. Using the same simulation setup (Fig. 3), we investigate this issue for the cases of defective metasurface with one cavity (Fig. 4(a)) and two cavities (Fig. 4(c)). Figure 4(b) and 4(d) show the field intensity on the *xz* plane along A-A′ and B-B′. For both cases, we observe that the field intensity is relatively low in the non-cavity region surrounding the hotspot. The low intensity is attributed to three physical mechanisms: 1) due to the different resonant frequency, there is little power coupled from the Tx resonator (operating at *f*_0_) to the non-cavity regions of the metasurface (operating at *f*_loc_), 2) because of the deep hybridization bandgap, there is only small leakage from the hotspot to the non-cavity region, 3) the Rx resonator and probe are closely coupled to the cavity region of the metasurface. The magnetic field is correlated to the scattering parameters, as explained in the Methods section. Because the proposed metasurface is operating in the near-field regime where the coupling is realized using non-radiative fields, the results indicate that the power reflected by the metasurface can be neglected.

### Control of location, shape, and intensity

**Figure 5:**
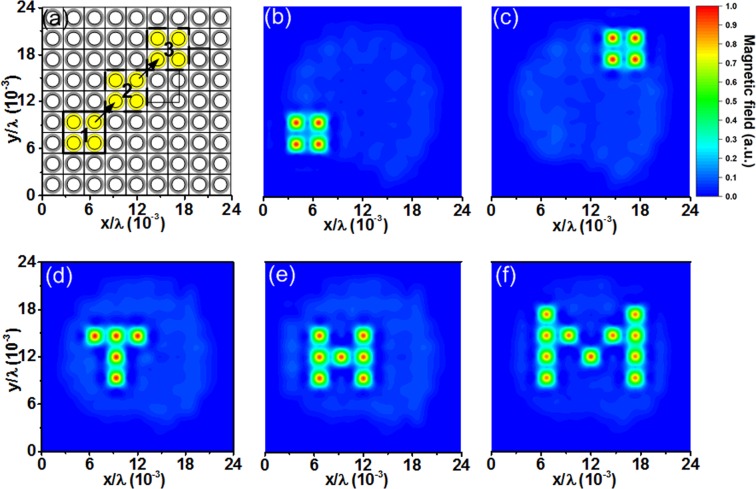
(**a**) Controlling the location of a field-focused region or hotspot. The cavity is created by activating four nearby unit cells. The desired path for moving the hotspot is indicated by arrows from 1 to 3. Field intensity distributions at (**b**) Location 1 and (**c**) Location 3. Measured field distributions showing the shapes of (**d**) T, (**e**) H, (**f**) M.

Next, we demonstrate that the location, shape, and intensity of the field-focusing region can be controlled precisely as desired. Figure 5(a–c) show the location control of the hotspot. We form a square cavity and reposition it through the desired path indicated by arrows from 1 to 3, as shown in Fig. 5(a). Figure 5(b,c) show the repositioning of the cavities along the desired path. These results demonstrate that the cavities can be freely positioned on the metasurface as desired. To demonstrate the ability to control the cavity shape, we form the letters T, H, and M. The measured results are shown in Fig. 5(d–f). By selectively activating cells in different shapes, the field can be localized into those letter shape regions.

**Figure 6:**
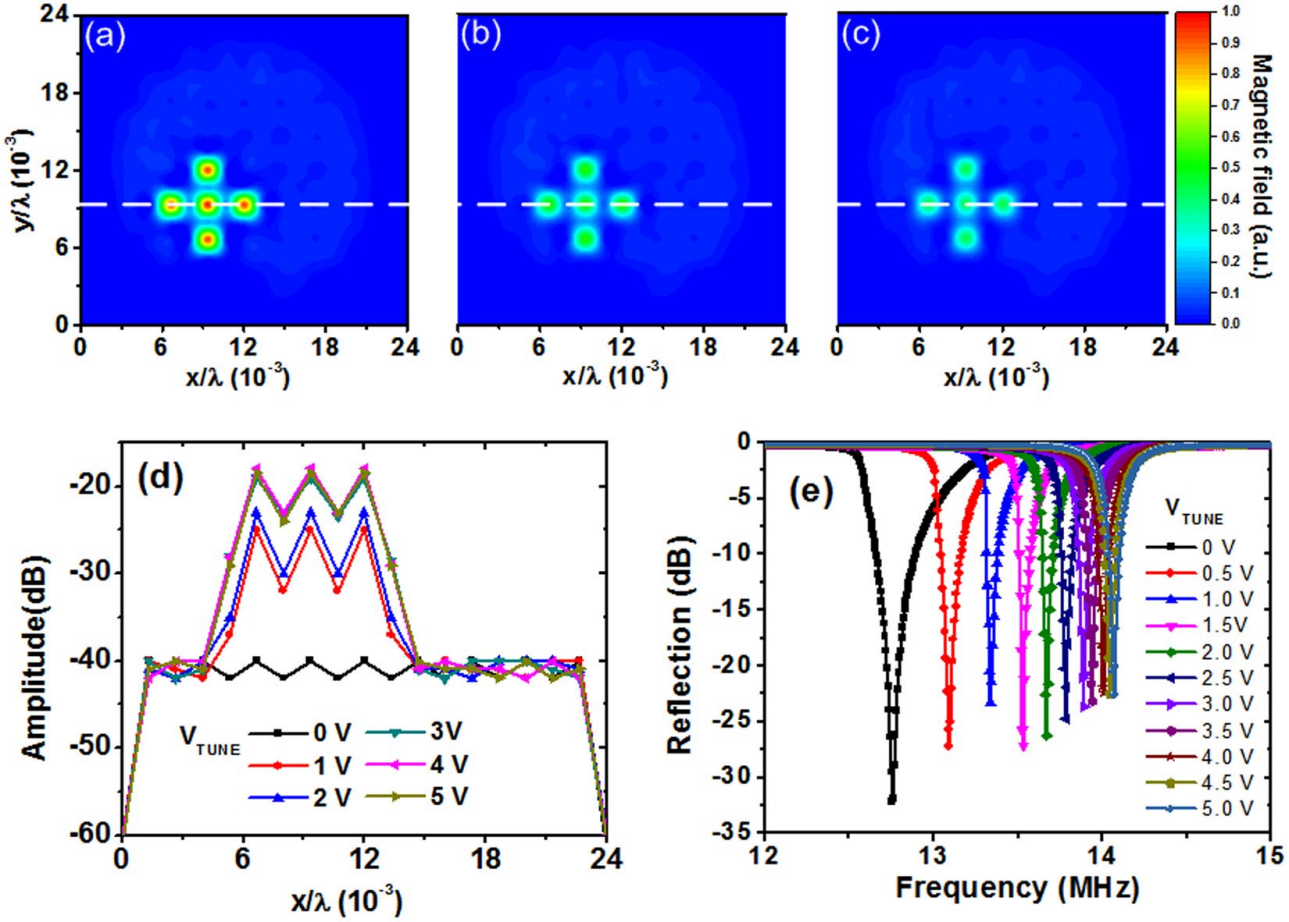
Measured field distribution for *V*_TUNE_ of (**a**) 5 V, (**b**) 2 V, (**c**) 1 V. (**d**) Measured transmission at *z* = 19.5 cm along the *x*-axis. This result demonstrates intensity control using cavities. (**e**) Resonance tuning characteristics.

For power control, we investigate a method to control the intensity of the field-focusing region. Figure 6(a–c) show the measured field intensity. A ‘+’ shape is created, demonstrating the different intensities. The intensity control is realized by tuning voltages *V*_TUNE_ and does not require a structural change to the metasurface. To show a quantitative value, we obtain the field amplitude along the *x*-axis at 9.2/λ × 10^−3^, which is indicated by a line. Figure 6(d) shows the measured transmission for different *V*_TUNE_, with a 21-dB dynamic range in the cavity region. Figure 6(e) shows the measured resonance tuning characteristics for different values of *V*_TUNE_. As *V*_TUNE_ increases, it shifts the resonance frequency from 12.8 to 14.1 MHz. Through that resonance de-tuning, the transmission characteristic of the cavity changes, thereby controlling the amount of transferred power. The results show the potential of the proposed method for fine intensity control of the hotspot.

### Wireless power routing

By dynamically reconfiguring the locations of the cavities, we create wireless power routing paths. This approach is slightly different from the original setup (Fig. 1(a)) using a relatively large Tx coil to expose the metasurface and selectively control the transmission. In power routing, a small Tx coil suitable for a point source is used to supply power to a unit cell. The routing paths provide wireless power from single/multiple Tx to multiple Rx locations on the metasurface. Using the reconfigurability of the active metasurface, various paths can be created by activating a line of cavities. For efficient power guidance, good field confinement is desirable. To characterize the power routing path, we measure the field intensity distributions. Cavities are created along the linear path in the center of the defective metasurface. The Tx coil is located at the center of the left *y*-axis (i.e., *x* = 0, *y* = 12/λ× 10^−3^), and the Rx probe scans the metasurface.

**Figure 7:**
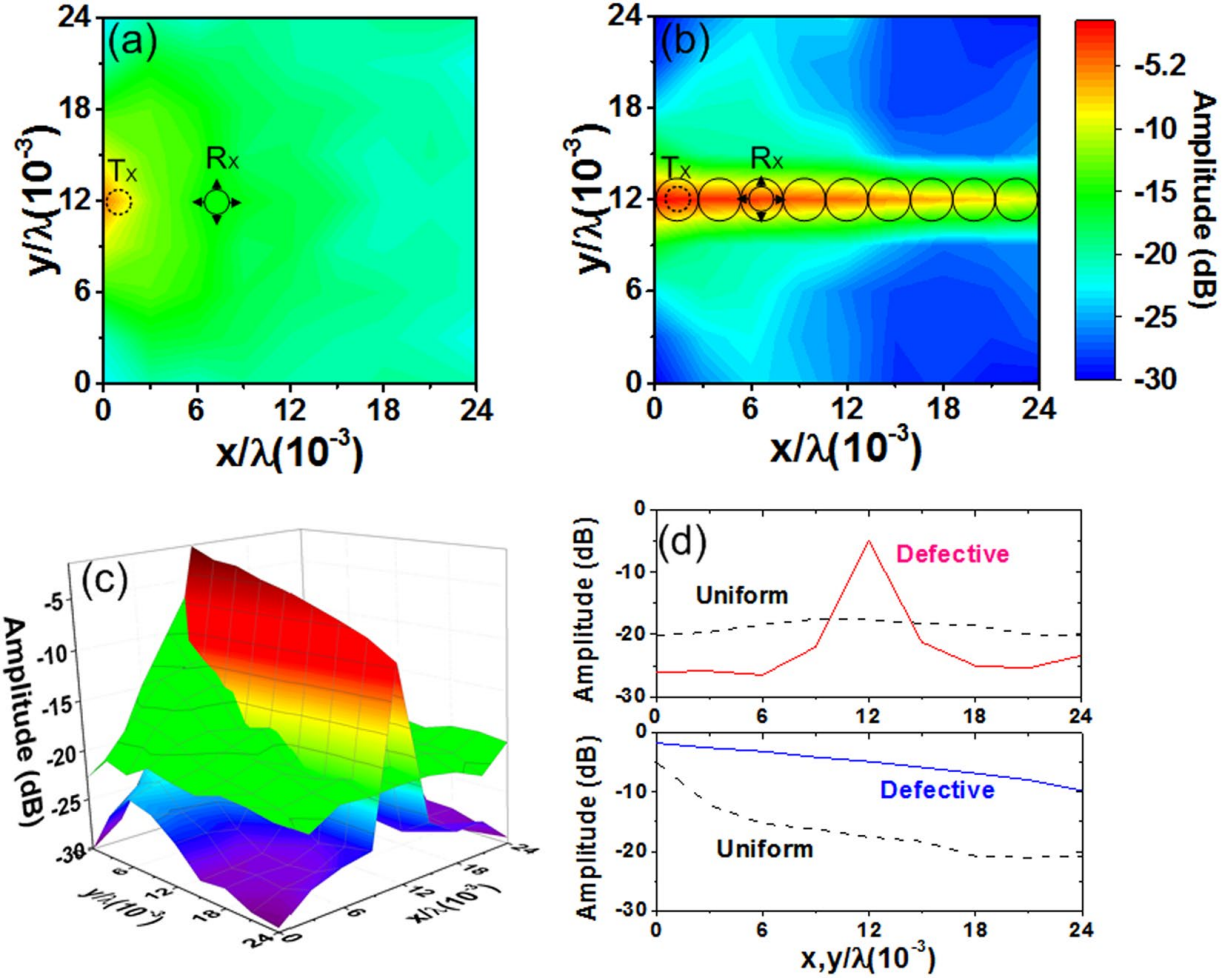
Measured field amplitude of (**a**) uniform metasurface, (**b**) defective metasurface with cavities forming a linear path. (**c**) Comparison of field amplitude where two results are superimposed. (**d**) Comparison of field amplitude measured along the *x-* and *y*-axes.

Figure 7(a,b) show the field distributions for uniform and defective metasurfaces, respectively. The results show the enhanced waveguiding characteristic provided by the defective metasurface. Figure 7(c) compares two cases using a bird’s eye view. On the uniform metasurface, the field spreads over a relatively broad area. On the defective metasurface, fields are localized along the waveguide and suppressed elsewhere. Figure 7(d) compares the field amplitude along the center of the *x-* and *y*-axes. With a uniform metasurface, the amplitude is distributed relatively evenly (about −20 dB) along the *y*-axis. With a defective metasurface, there is a peak value at the center (about −5 dB), with suppression along the other locations (about −25 dB). For the uniform metasurface, the amplitude decreases from −5 to −21 dB along the *x*-axis. For the defective metasurface, it varies from −1.6 to −9.8 dB. Thus, the defective metasurface provides enhanced field confinement along the path.

**Figure 8:**
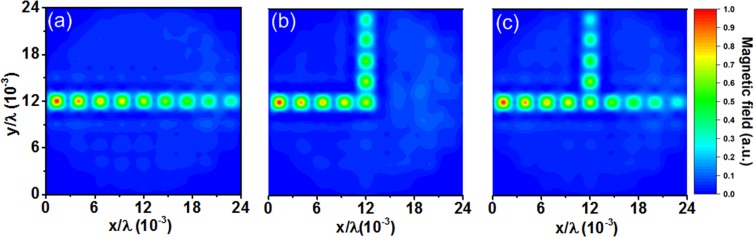
Wireless power routing paths formed by a line of cavities on the defective metasurface: (**a**) linear, (**b**) bent, and (**c**) split paths.

Using the same experimental setup (Fig. 7), we demonstrate three types of routing paths: linear, bent, and split, as shown in Fig. 8(a–c). These paths provide convenient ways to route wireless power to arbitrary cell locations on the metasurface. The split path provides an evenly distributed power division between two ports. The field intensity of the bent path is similar to that of the linear path, demonstrating a low loss method for bending surface waves. This is in contrast to the waveguide formed using spoof surface plasmons, in which a bent path usually incurs a relatively high loss through backscattering and radiation. The results show that the subwavelength dimension of the unit cells allows wireless power guiding beyond the conventional wavelength limit. This approach thus provides dynamically reconfigurable wireless power routing, which is an obvious advantage over the passive approach, which requires a structural change to create different routing paths^26^.

**Figure 9:**
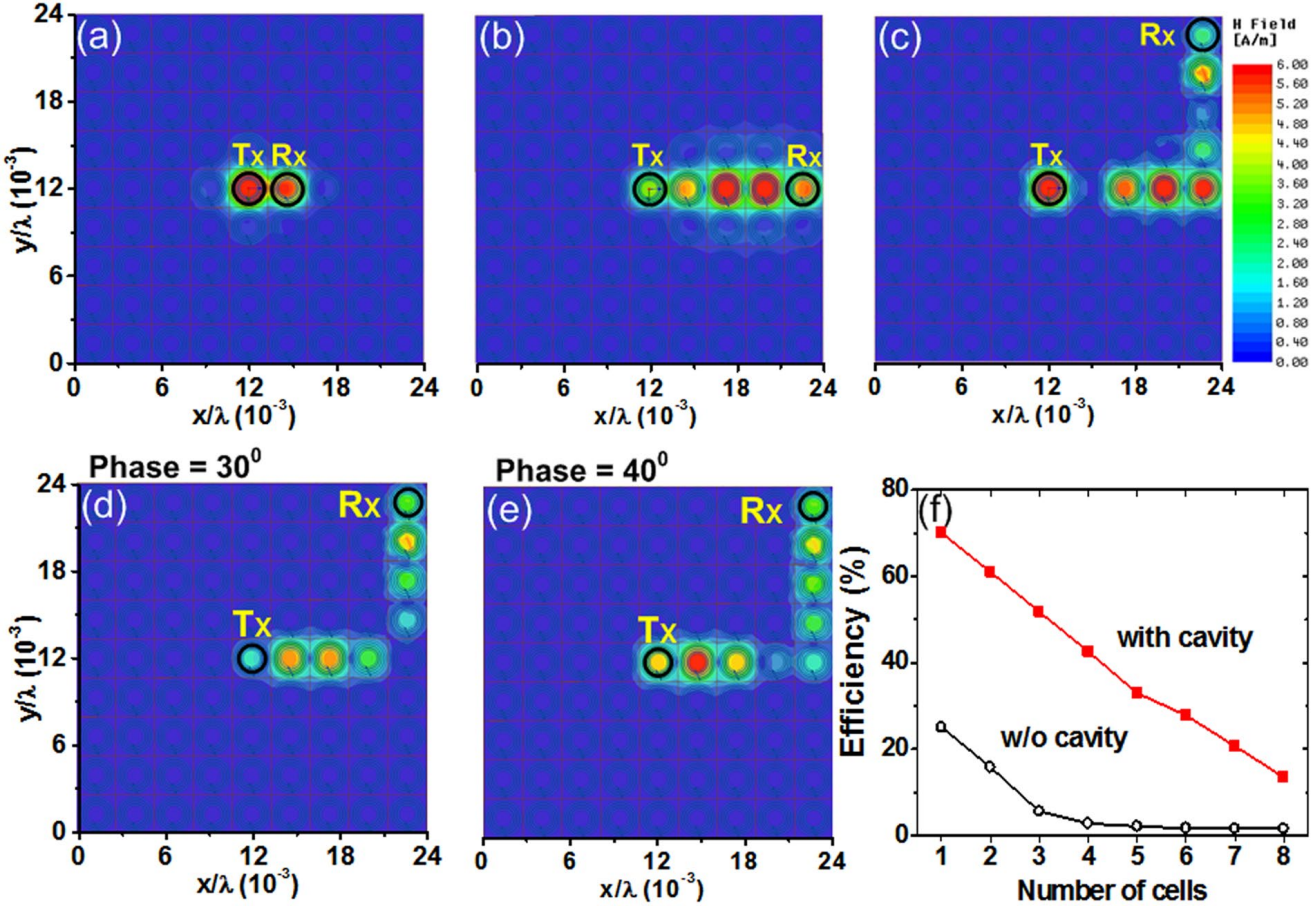
Field distribution obtained using EM simulations for different numbers of cells: (**a**) two, (**b**) five, and (**c**) nine. Field distributions for the bent path at two phases, (**d**) 30° and (**e**) 40°. (**f**) Measured efficiencies as a function of the number of cells apart. In the case of one cell, the Tx and Rx probes are located at the opposite sides of the metasurface.

To investigate energy hops from one cavity to the next, we use the EM simulator. For the bent path, we obtain results for different numbers of cells. Figure 9(a–c) show the field distributions for two, five, and nine cells. The coupling between successive cells allows the field to propagate along the desired path. Figure 9(d,e) show the field distributions at 30° and 40° when power is sent from the Tx to Rx cells. With lateral coupling between cells, we observe backward wave propagation^28^. The efficiency for a different set of cells is shown in Fig. 9(f). Starting from the Tx cell, we measure efficiency while moving the Rx cell along the bent path. When a cavity is used, the peak efficiency between two cells is as high as 70.1%, and it is 33.3% for five cells. Compared with the uniform metasurface, this corresponds to improvements of 2.8× and 15.1×, respectively. This result shows the potential of the dynamically reconfigurable power routing paths created using cavities, which can deliver power to a specific cell on the metasurface.

## Discussion

Maznev *et al*. investigated the phenomenon when propagating waves interact with a 2D array of local resonators on a metasurface^36^. A bandgap appears between the bulk and surface wave propagations. *Below* the resonance of local oscillators, surface waves (or guided mode) exist. When the frequency approaches the resonance, the dispersion characteristic gets flattened, and the mode becomes increasingly localized (See Fig. 1(b)). The magnitude of the wave vector significantly exceeds that of the free space. This provides suitable conditions for sub-diffraction imaging and focusing. This approach has been used to beat the diffraction limit for acoustic^40–42^ and microwave applications^43^.

The defect cavity mode investigated in this work is somewhat different from the previous approach, which uses flat dispersion *below* the local resonance. Our cavity mode is created *above* the flat dispersion where a bandgap is located. In the case of the propagating wave, this *above* region is a bandgap, and propagation is not allowed. However, we design the metasurface using a suitable resonance frequency and coupling coefficient so that MIWs can propagate in this region (See Fig. 1(b)). Considering only the coupling between nearest neighbors and neglecting the loss, the dispersion characteristic of the MIWs can be expressed as

1 $$\beta =\frac{1}{a}\arccos [\frac{{\omega }_{0}^{2}/{\omega }^{2}-1}{\kappa }]$$

where *β* is the wave number, *κ* is the coupling coefficient, and *a* is the lattice constant^37,44^. This shows that the dispersion differs from that of the spoof surface plasmons, which start at the light line and approach zero group velocity at the band edge. The dispersion of MIWs shows a cosine shape in the passband, as shown in Fig. 1(b), which is bounded by sharp stopbands at both ends of the passband^28^. The stopband is created by the increased loss toward the band edge.

The MIWs are a kind of slow wave supported by inter-element coupling^45^. The MIWs propagate on the surface, but they differ from the conventional guided mode^36^ and spoof surface plasmons^46^. The MIW propagation is similar to that of a coupled resonator optical waveguide (CROW)^47^ or a coupled-defect surface waveguide (CDSW)^48^. Similar to the CDSW, MIWs propagate though weak coupling between otherwise highly localized defect cavities. The MIW differs from CROW and CDSW in that the propagation is supported by magnetic coupling.

The cavity mode (or trapped mode) is a special form of MIW that is confined within a defect. Outside the cavity region, wave propagation is inhibited because the frequency is selected to fall within the bandgap. Therefore, the field is localized in the cavity region. At both sides of the passband where the MIWs propagate is a sharp stopband, which is attributed to high attenuation^28^. Because it is formed by Fano-type interference, the stopband is a deep bandgap. The Fano-type interference occurs between the incoming continuous fields and waves of discrete resonance re-excited by local resonators. Around the resonance frequency, the wave emitted by the resonator shows a steep phase change that creates a zero transmission or a deep hybridization bandgap. We note that the concept of MIWs is compatible with a microscopic approach to bandgaps^29^.

The use of a defect cavity has been successful in photonic crystals that control light by inhibiting wave propagation. That success has led to many important applications in optics. Because wave propagation is governed by an interference effect from medium periodicity, however, the application of photonic crystals has been limited mainly to optical frequencies. On the contrary, metamaterials are created by resonant cells and characterized by their subwavelength characteristics. The local modification does not change the overall macroscopic parameters of a metamaterial^49^. Spoof surface plasmons exhibit a subwavelength property, and a point defect mode created within a bandgap is used to demonstrate the subwavelength localization of the electric field^50^. Because the underlying physical mechanism is based on Rayleigh-Bloch waves, the mechanism differs from the Fano interference. Thus, introducing a defect into a metamaterial has a different effect from that of a photonic bandgap or spoof surface plasmons.

For the proposed active metasurface, we summarize the performance in terms of field localization capability, tuning speed, power transfer area, and efficiency of the overall system. The Purcell factor can be used to quantify energy coupling in the cavity mode and thereby the field localization capability^51^. Considering the 2D nature of the metasurface, we use the Purcell factor *F*_P_ for surface plasmonic polaritons, which can be expressed as

2 $${F}_{{\rm{P}}}=\frac{{{\Gamma }}_{{\rm{cav}}}}{{{\Gamma }}_{0}}\cong \frac{3}{4\pi }\frac{{\lambda }^{2}{n}_{{\rm{g}}}}{{A}_{{\rm{eff}}}}$$

where *Г*_cav_ is the decay rate of the cavity, *Г*_0_ is the decay rate in a vacuum, and *n*_g_ is the group index of the mode. The *A*_eff_ is the mode effective area, which is a measure of the spatial confinement in the cavity. Thus, *F*_P_ is determined by the group index and its transverse confined area. The MIWs propagating on the metasurface are a slow wave with a very large *n*_g_. Using the group velocity associated with the dispersion of MIWs^52^, we obtain *n*_g_ = 1980 at 14.1 MHz. Using *A*_eff_ = λ^2^/139000 in (2), we obtain a relatively high *F*_P_ = 6.58 × 10^7^. This result agrees with the theoretical Purcell factor^49^, which is obtained using a lattice spacing of 0.057 m and a wavelength normalized value of 0.0027.

The tuning speed for creating different cavity configurations is related to the switching time between two resonant frequencies, *f*_loc_ and *f*_0_. Because *V*_TUNE_ is connected to a varactor, which draws negligible current, the switching can be performed within nanoseconds, which is fast enough for most power applications. The minimum size of the power transfer area is determined by the size of the unit cell, which is a deep subwavelength scale of 2.7λ  ×  10^−3^. Using microfabrication processes, this can be further reduced. The power transfer efficiency of the device as a whole is described in detail in our previous publication, which does not include a tuning element^30^. The effect of various parameters on efficiency, which includes the size of the power receiving area, is discussed with experimental results. Because the power consumption of the tuning element is negligible, the efficiency of this work is not affected by the active tuning function. In the case of the size ratio of 16:1 between the Tx resonator and Rx probe, the proposed active metasurface achieves a peak efficiency of 50.4% which is significantly higher than the efficiency 7.8% of free space. Our metasurface works at near field regime in the MHz frequency range. In addition to multi-receiver WPT, the proposed approach can be applied to enhanced MRI systems, near field focusing, and subwavelength devices.

## Methods

### Metamaterial design and fabrication

A metasurface consisting of 9 × 9 arrays of unit cells is realized using four-turn spiral resonators (4T-SRs). Schematics of the 4T-SR and tuning element are shown in Fig. 1(a). They are fabricated using a standard printed circuit board (PCB) process on FR-4 substrate (relative permittivity = 4.4, loss tangent = 0.02). The dimensions are as follows: size of unit cell = 5.7 × 5.7 cm^2^, copper strip width = 3 mm, inter-strip spacing = 2 mm, outer radius = 28 mm, thickness of copper strips = 0.1 mm, and substrate thickness = 1 mm. The 4T-SR is designed to achieve a high *Q*-factor. Because the intrinsic gap capacitance of a spiral is inadequate, we add *C*_0_ = 220 pF (Samwha CS1608) to make the unit cell resonate at a lower frequency. A resistor *R*_1_ = 51 Ω (Vishay RCP0505) is used for current limiting. The capacitance of the varactor *C*_VAR_ (Skyworks SMV1255-079LF) is controlled by the tuning voltage *V*_TUNE_. In addition to determining the resonance frequency, two capacitors, *C*_1 = _*C*_2 = _1 nF (Wurth Elektronik 0603), prevent a short-circuit by *V*_TUNE_. Two inductors, *L*_1_ = *L*_2_ = 100 μH (TDK MLF2012), provide isolation between unit cells. This isolation is needed to separately control the resonant frequency of each unit cell. We characterize the isolation between adjacent unit cells using a multimeter (5.1 MΩ at DC) and a network analyzer (32.4 dB at 14.1 MHz). The unit cell design eliminates power dissipation except for nA leakage current in the tuning circuitry.

### Active metamaterial operation

To create a hotspot, the cells surrounding the cavity, which create the hybridization bandgap (Fig. 1(d)), resonate at the local resonance frequency *f*_loc_. An upward frequency shift to *f*_0_ allows their resonances to fall within the hybridization bandgap. The resonance frequency of the unit cell varies according to the capacitance of the series combination of *C*_VAR_, *C*_1_, and *C*_2_. The values of *C*_VAR_ at *V*_TUNE_ = 0 and 5 V are 50 and 5 pF, respectively. Using the two *C*_VAR_ values, the active metasurface provides switchable functions between two distinct frequencies, as shown in Fig. 1(c). Using *V*_TUNE_ = 0 V, the unit cell resonates at *f*_loc_ = 12.8 MHz. When the value of *C*_VAR_ is reduced using *V*_TUNE_ = 5 V, the unit cell resonates at *f*_0_ = 14.1 MHz.

### Transmission measurement

To obtain a field intensity distribution, we make non-invasive measurements of scattering parameters using a small loop as an Rx probe. A non-resonant loop reduces the disturbance on unit cells, and thus it does not significantly affect their original resonance frequency. The axis of the Rx probe is kept normal to the metasurface. Therefore, it is sensitive to the magnetic field perpendicular to the metasurface. The distance between the Tx coil and metasurface is 15 cm. The transmission is measured using a network analyzer (Keysight 5063 A). We scan the whole metasurface on the *xy* plane at *z* = 0.5 cm at the desired frequency with 361 data points. We set the intermediate-frequency (IF) bandwidth of the network analyzer to 500 Hz to adequately resolve the weak signals. Due to the correlation between the scattering parameters and the magnetic field^53^, the spatial distribution of the magnetic field can be drawn in terms of the transmission coefficient, *S*_21_^29^. The relative magnitude of the magnetic field is the primary concern because our objective is to identify the relative intensity difference between the cavity and the neighboring regions.

### Efficiency measurement

Resonator coupling is used for the power efficiency data. The transmitter consists of a Tx coil and a Tx resonator. The receiver consists of an Rx resonator and an Rx probe. The Tx coil is a one-turn loop with a diameter of 25 cm. The Tx resonator has a turn of 5, a pitch of 1 cm, and an inner diameter of 30 cm. The radius of the Rx resonator is about one-eighth smaller than that of the Tx resonator. The Rx probe, which is about half the size of the Rx resonator, is a one-turn loop with a diameter of 3 cm. Thus, the size ratio of the Tx resonator and Rx probe = 16:1. The small dimension is chosen to effectively couple the field localization realized in the cavity. For the efficiency measurement, *S*_21_ is obtained using a network analyzer following the short-open-load-through (SOLT) two-port calibration method. To mitigate the effects of impedance mismatch, both port-1 and port-2 are matched^54^. Thus, the power transmission efficiency can be estimated using |S_21_|^2^.

### EM simulations

All the simulation results are obtained using a commercially available, finite element method solver–based EM simulator, Ansys HFSS. The magnetic field distribution obtained with the EM simulator is compared with the measurements to confirm the results. The *Q*-factor is obtained using the 3D parasitic extractor Ansys Q3D and the expression *Q* = (*ωL*)/*R*. The reported *Q*-factor at 14.1 MHz involves the lumped components.
